# Changes in the Emergency General Surgery Operations in the Setting of COVID-19 and Impact of Strategy of Non-Operative Management on Outcomes in Acute Appendicitis

**DOI:** 10.7759/cureus.27552

**Published:** 2022-08-01

**Authors:** Muhammad S Ahmad, Iannish Sadien, Bogdan Ivanov

**Affiliations:** 1 Upper GI Surgery, Worcestershire Acute Hospital National Health Service (NHS) Trust, Worcestershire, GBR; 2 General Surgery, The Princess Alexandra Hospital National Health Service (NHS) Trust, Harlow, GBR

**Keywords:** emergency surgery, normal histology, negative appendicectomy, non-operative, acute appendicitis, covid-19

## Abstract

Background: Coronavirus disease 2019 (COVID-19) has posed significant challenges to the provision of elective and emergency general surgical care. Patterns of presentation have changed and management pathways have also been adapted, moving to more non-operative management (NOM) for some conditions. We investigated how COVID-19 changed the volume of emergency general surgery operating in our district general hospital (DGH). We aimed to evaluate the impact of NOM on outcomes in acute appendicitis.

Methods: A retrospective case review of operating lists, patient handover lists, and patient notes was undertaken for patients presented between 1^st^ January 2020 and 3^rd^ June 2020. The study period was divided into two, with the period between 1^st^ January 2020 and 23^rd^ March 2020 representing the pre-COVID cohort.

Results: Some 393 emergency general surgery operations were performed in the study period. There was a clear reduction in operating volume after 23^rd^ March 2020. During that same period, 325 patients were assessed with right iliac fossa (RIF) pain. Median age was 21 (range 5-87) and 201 patients were female (61.8%). The rate of NOM for suspected acute appendicitis was 8.8% in the pre-COVID group, which increased to 36.4% in the COVID group. The incidence of normal histology following appendicectomy did not change with this difference in management (16.1% compared to 17.9%, p = 0.78).

Conclusions: This study summarizes the changes brought to the provision of emergency general surgery in the setting of a DGH by the COVID-19 pandemic. In particular, NOM was the preferred option for acute appendicitis but this did not alter the negative appendicectomy rate.

## Introduction

Coronavirus 2019 (COVID-19) was declared a global pandemic on 11th March 2020 by the World Health Organization [1]. The first confirmed COVID-19-related death in the United Kingdom was on 5th March 2020 and an acute rise in cases nationally led to the imposition of a national lockdown on 23rd March 2020 by the British government [2-3]. Uncertainty about the mode of transmission and best preventative measures led to significant rethinking and reorganization of usual elective and emergency general surgical pathways [4-5]. Non-urgent elective operations were postponed to reduce the strain on intensive care capacity [6]. The traditional management of some common emergency general surgical presentations was also adapted [7-8].

Non-operative management (NOM) for conditions such as acute appendicitis or acute cholecystitis was advocated [8]. Operative management was reserved for patients who failed conservative management or were deemed too unwell to avoid surgery. In addition to this, the safety of laparoscopy was questioned due to concerns about aerosol generation and potential propagation of severe acute respiratory syndrome coronavirus 2 (SARS-CoV-2), and a strategy of open surgery was advocated as much as possible [8-9]. These drastic changes to the management of general surgical emergencies could have a significant impact on patient outcomes but also long-term effects on how surgical departments throughout the United Kingdom operate over the next few months.

We aimed to evaluate how the emergency general surgery workload changed with the COVID-19 pandemic in our district general hospital (DGH). We then wanted to investigate whether an approach of NOM for acute appendicitis altered outcomes. In particular, our hypothesis was that this approach would reduce the rate of normal histology following appendicectomy.

## Materials and methods

Study design

This was a retrospective case review performed in a DGH in the United Kingdom. Patients presenting between 1st January 2020 and 3rd June 2020 and who underwent a general surgical operation in that time period were identified retrospectively from the electronic theater record system.

Patients included and data collected

Patients presenting with right iliac fossa (RIF) pain during the period from 1st January 2020 to 3rd June 2020 were identified using local on-call general surgery handover lists. Patient information such as baseline demographics and suspected diagnosis on presentation was collected. For patients who underwent surgery for RIF pain (diagnostic laparoscopy, laparoscopic appendicectomy, or open appendicectomy), the final diagnosis was obtained from the operation note or from histology of the resected specimen on the electronic results reporting system. Post-operative complications were noted from the discharge summaries and patient notes and reported using the Clavien-Dindo classification [10]. To aid analysis, the study period was divided into two. Patients presenting or operated on before 23rd March 2020 were in the pre-COVID group, while patients after this date were in the COVID group.

Statistical analysis

The mean with standard deviation (SD) or median with interquartile range (IQR) was used to describe continuous variables. A t-test was used to compare means for continuous data between two groups while Fisher’s exact test was used for categorical variables. A two-tailed p-value of 0.05 was assumed to be the threshold of statistical significance. Analysis was by intention-to-treat. All analyses were performed using R version 3.4.4 (R Foundation for Statistical Computing, Vienna, Austria) using tidyverse and ggplot2 packages.

Ethical approval

Local hospital ethics committee exempted this study from ethical approval and was registered as an audit.

## Results

Total number of emergency operations (general and vascular surgery only)

**Figure 1:**
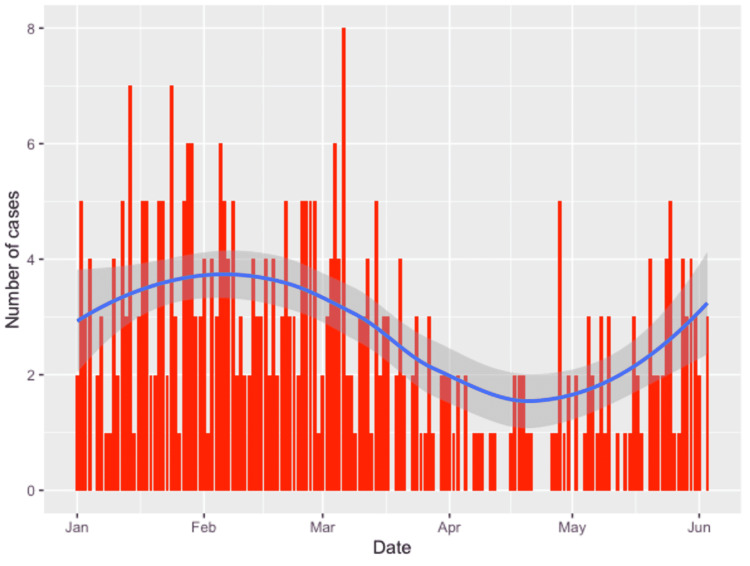
Daily emergency surgery volume by month.

There were a total of 381 emergency operations between January and June 2020. The median age was 43 years (range 5-93 years). The daily emergency surgery volume variation by month is shown below (Figure 1).

**Figure 2:**
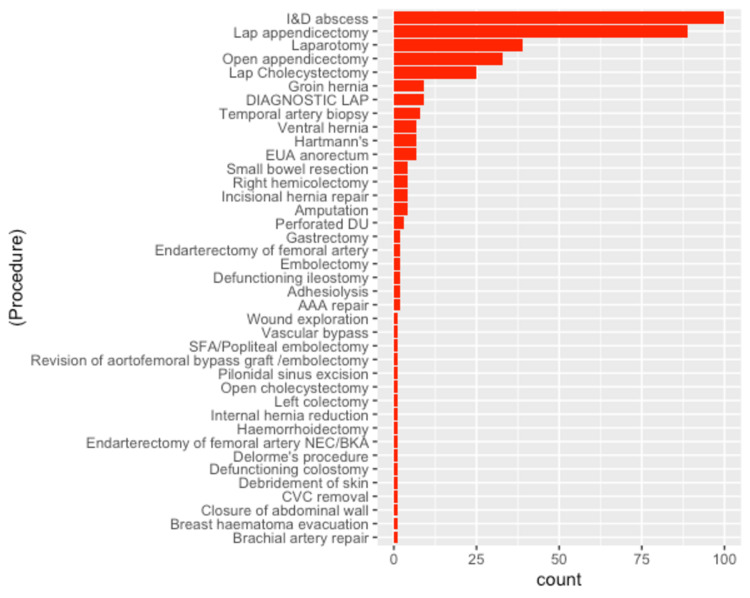
Operations performed during study period.

The breakdown of operations performed in that time period is shown below (Figure 2).

**Figure 3:**
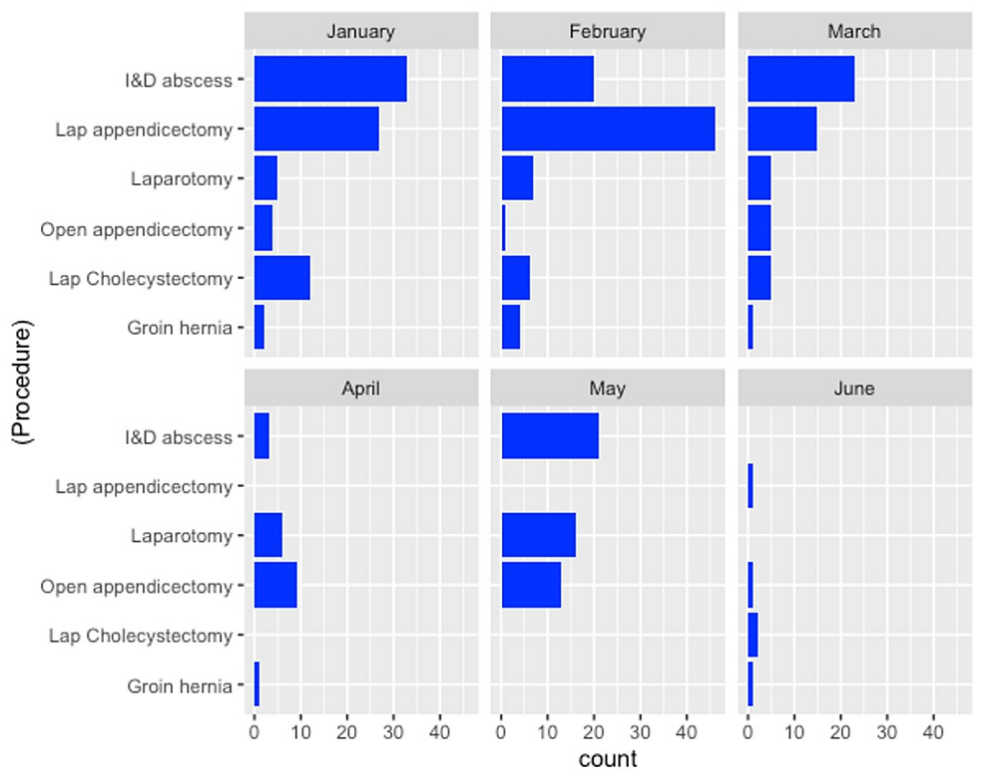
Most common operations over time.

Prior to the onset of COVID-19, the majority of appendicectomies were performed laparoscopically but following a local policy change, open surgery was the preferred approach after 20th March 2020 (Figure 3).

Right iliac fossa pain

**Figure 4:**
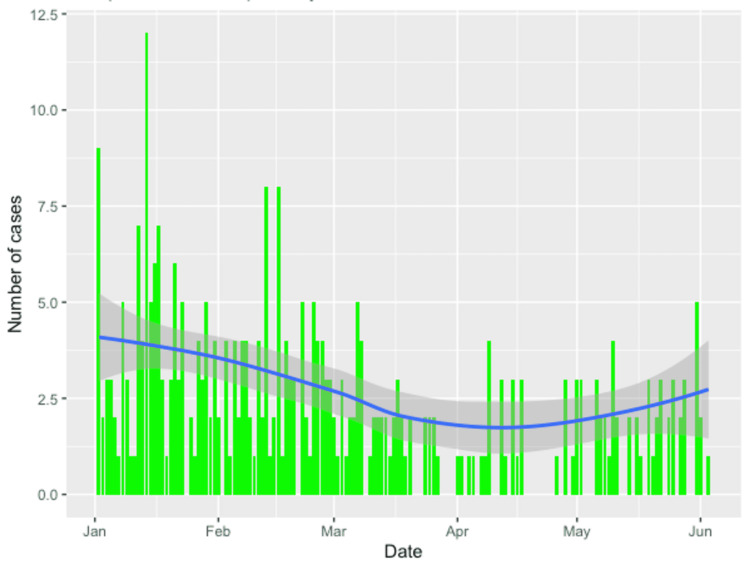
Daily variation in the number of RIF pain presentations. RIF, right iliac fossa

Some 325 patients were seen with RIF pain between 1st January and 3rd June 2020. The daily variation of presentations is shown below (Figure 4).

Patient Characteristics

The median age was 21 (range 5-87) and 201 patients were female (61.8%). The three most common suspected diagnoses at presentation were acute appendicitis, non-specific abdominal pain, and mesenteric adenitis (Table 1).

**Table 1:** Most common suspected diagnoses for RIF pain. RIF, right iliac fossa

	Acute appendicitis	Non-specific pain	Mesenteric adenitis	Ovarian cyst	Pelvic inflammatory disease
Number (percentage)	146 (44.9%)	84 (25.8%)	32 (9.8%)	12 (3.7%)	5 (1.5%)
Median age (range)	21 (5-86)	19.5 (5-61)	9.5 (5-24)	21 (10-31)	30 (19-63)
Male/female	64/82	27/57	19/13	0/12	0/5

Concerning patients with suspected acute appendicitis, a total of 115/146 (78.7%) were diagnosed radiologically by CT and ultrasound scans, while the rest 31/146 (21.2%) were diagnosed clinically and by diagnostic laparoscopy.

Management: Surgery Versus Conservative and Associated Complications

A total of 25/146 (17.1%) patients with suspected appendicitis were managed with conservative intent, while the rest 121/146 (82.8%) underwent primary operative management. Of those who had surgery in the pre-COVID period, 82/88 (93.1%) patients underwent laparoscopic appendectomy, compared to 8/33 (24.2%) in the COVID period. The rate of laparoscopic and open appendectomies is shown below (Table 2).

**Table 2:** Rate of laparoscopic and open appendectomies during COVID and pre-COVID.

	Pre-COVID period	COVID period
Laparoscopic appendectomy	82	8
Open appendectomy	6	25

In pre-COVID era, 9/102 (8.8%) patients were managed conservatively, compared to 16/44 (36.4%) in the COVID period (Fisher’s exact test, p = 0.0002). Conservative management failed in 3/9 (33.3%) patients in the pre-COVID era, compared to 2/16 (12.5%) during COVID (p = 0.3123).

Of those who had surgery in the pre-COVID period, there were 13/93 (14.0%) complications, compared to 4/28 (14.3%) patients in the COVID era (p = 1.0). A breakdown of complications is shown below (Table 3).

**Table 3:** Breakdown of post-operative complications.

	Pre-COVID period	COVID period
Clavien-Dindo I	1	1
Clavien-Dindo II	7	3
Clavien-Dindo III	4	0
Clavien-Dindo IV	0	0
Clavien-Dindo V	1	0
Total	13 (14%)	4 (14.3%)

One patient who had a laparoscopic appendicectomy in the pre-COVID period developed a leak from the appendicular stump site, which required a return to the theater and subsequent right hemicolectomy. The patient had a prolonged stay in hospital and subsequently developed COVID-19 pneumonia, from which they succumbed.

Negative Appendicectomy Rate

Of 93 appendicectomies performed in the pre-COVID period, 15 showed normal histology (16.1%). This compares to 5/28 (17.9%) in the COVID period (p = 0.7791). The remaining histology is shown in the table below (Table 4).

**Table 4:** Histology of patients undergoing appendicectomy.

	Pre-COVID period	COVID period
Acute appendicitis	69 (74.1%)	23 (82.1%)
Normal	15 (16.1%)	5 (17.9%)
Enterobius vermicularis	7 (7.5%)	-
Neuroendocrine tumor	2 (2.1%)	-

## Discussion

Our study reports our local experience with emergency general surgery workload during the COVID-19 pandemic and compares it to the immediately preceding months. We report that the volume of cases saw a drop in April but has steadily increased to reach pre-COVID levels. This ‘lockdown effect’ can be explained by patients not wanting to attend the hospital from fear of contracting SARS-CoV-2 during the few weeks of the national lockdown [11]. It is interesting that this did not lead to a rebound of complex surgical cases representing delayed presentations. One possible explanation could be an increase in antibiotic therapy in primary care for conditions that would have otherwise led to hospital presentation and possible surgery, such as acute appendicitis, acute cholecystitis, or abscesses [12].

Following a change in departmental policy based on national guidance issued on 20th March 2020, the percentage of patients with suspected acute appendicitis who were treated non-operatively increased from 8.8% pre-COVID to 36.4% in the COVID period. Interestingly, this did not lead to a reduction in the negative appendicectomy rate (16.1%-17.9%). One of the reasons could be that the use of open surgery compared to laparoscopy may have impeded adequate exploration of the RIF to exclude other causes of RIF pain such as ovarian pathology or mesenteric adenitis [13]. Surgeons who perform an open appendicectomy may then be more likely to remove a normal-looking appendix to justify the decision to operate. This is in contrast to the laparoscopic approach where finding an alternative diagnosis usually means that a normal appendix can be left in situ. It is worth noting, however, that the complication rate following the open approach was similar to that using laparoscopy.

Conservative management of acute appendicitis failed in 33.3% of the pre-COVID group compared to 12.5% in the COVID group. While this difference did not reach statistical significance, a possible explanation could be that surgeons were more likely to continue conservative measures to avoid an operation during the COVID period as much as possible. This could have involved prolonged or escalated antibiotic therapy [14].

The complication profile following appendicectomy in the pre- and COVID groups did not differ. This is surprising considering that the vast majority of appendicectomies were performed laparoscopically prior to 20th March 2020 in contrast to the open approach after that date. In fact, published literature suggests that open appendicectomy is associated with increased wound infection rates [15]. While we did not observe this in our study, it is possible that patients with a wound infection self-cared or sought help from primary care, and therefore, did not attend secondary care.

Our study is not without limitations. First, the retrospective nature of the data means that it is prone to selection bias. Indeed, we do not have information on patients with other acute conditions such as acute cholecystitis who were managed conservatively. This is one of the reasons why we decided to limit our sub-analysis to acute appendicitis. Furthermore, we were only able to capture the management of general surgical patients in secondary care only. As previously mentioned, a proportion of cases may well have been managed conservatively in primary care with analgesia and/or antibiotics given the COVID-19 pandemic. However, this proportion is likely to be small and any eventual failure of conservative management would have been reflected in our data.

## Conclusions

In summary, we describe the change in the pattern in emergency general surgical operating in a DGH in the UK at the peak of the COVID-19 pandemic. We also demonstrate that a strategy of more NOM of acute appendicitis does not reduce the negative appendicectomy rate in the setting of COVID-19. This data will hopefully add to the growing body of literature on the impact of COVID-19 on emergency general surgery provision.
